# Novel brewing yeast hybrids: creation and application

**DOI:** 10.1007/s00253-016-8007-5

**Published:** 2016-11-24

**Authors:** Kristoffer Krogerus, Frederico Magalhães, Virve Vidgren, Brian Gibson

**Affiliations:** 1 VTT Technical Research Centre of Finland, Tietotie 2, P.O. Box 1000, 02044 Espoo, Finland; 2 Department of Biotechnology and Chemical Technology, Aalto University, School of Chemical Technology, Kemistintie 1, Aalto, P.O. Box 16100, Espoo, 00076 Finland

**Keywords:** Lager, *S. eubayanus*, Brewing, Mating, Heterosis, Aroma, Stress tolerance, Stability

## Abstract

The natural interspecies *Saccharomyces cerevisiae* × *Saccharomyces eubayanus* hybrid yeast is responsible for global lager beer production and is one of the most important industrial microorganisms. Its success in the lager brewing environment is due to a combination of traits not commonly found in pure yeast species, principally low-temperature tolerance, and maltotriose utilization. Parental transgression is typical of hybrid organisms and has been exploited previously for, e.g., the production of wine yeast with beneficial properties. The parental strain *S. eubayanus* has only been discovered recently and newly created lager yeast strains have not yet been applied industrially. A number of reports attest to the feasibility of this approach and artificially created hybrids are likely to have a significant impact on the future of lager brewing. De novo *S. cerevisiae* × *S. eubayanus* hybrids outperform their parent strains in a number of respects, including, but not restricted to, fermentation rate, sugar utilization, stress tolerance, and aroma formation. Hybrid genome function and stability, as well as different techniques for generating hybrids and their relative merits are discussed. Hybridization not only offers the possibility of generating novel non-GM brewing yeast strains with unique properties, but is expected to aid in unraveling the complex evolutionary history of industrial lager yeast.

## Introduction

Beer and other fermented beverages have been produced for thousands of years and have played an important part in most human societies (Hornsey 2003, 2012). Yeast (primarily of the *Saccharomyces* genus) play a vital role in beer production and quality; during fermentation, they not only convert wort carbohydrates into ethanol and CO_2_, but also synthesize various key flavor compounds. Traditionally, brewer’s yeasts have been divided into top- and bottom-fermenting strains depending on their fermentation behavior, but modern molecular techniques have revealed high diversity between yeast strains used for brewing (Gallone et al. 2016; Legras et al. 2007; Liti et al. 2005; Steensels et al. 2014). Moreover, many of the yeast strains that brewers have used for centuries have now been shown to be interspecific hybrids. In particular, lager yeast or *Saccharomyces pastorianus*, the workhorse of the modern brewing industry, is known to be an interspecific hybrid between *Saccharomyces cerevisiae* and the cold-tolerant *Saccharomyces eubayanus* (de Barros Lopes et al. 2002; Dunn and Sherlock 2008; Libkind et al. 2011; Liti et al. 2005; Nilsson-Tillgren et al. 1981; Tamai et al. 1998). In addition, natural hybrids between *S. cerevisiae* and *Saccharomyces kudriavzevii* have been isolated from Belgian Trappist beers (González et al. 2008), while natural hybrids between *S. cerevisiae* and *Saccharomyces uvarum* are used frequently in winemaking (Le Jeune et al. 2007). The hybrid state appears to confer a competitive advantage in the fermentation environment. In the case of the lager yeast, success has been due to a fortunate combination of phenotypes. Low-temperature fermentation was enabled by the inheritance of cryotolerance from *S. eubayanus*, while efficient maltotriose utilization and other beneficial fermentation properties were inherited from *S. cerevisiae* (Hebly et al. 2015; Krogerus et al. 2015). Despite the industrial importance of lager yeasts, much of their natural history remains obscure.

The hybrid nature of *S. pastorianus* had been suspected for some time. Early research, particularly from the Carlsberg Laboratory in Copenhagen, showed that the lager yeast genome included genetic material derived from *S. cerevisiae* and a non-*S. cerevisiae* yeast (Nilsson-Tillgren et al. 1981; Pedersen 1985; Hansen et al. 1994). As early as 1944, Øjvinde Winge had described the poor sporulation ability of lager yeast; an indication that they did not represent a pure species (Winge 1944). The exact composition of the hybrid in terms of parentage and ploidy did not begin to become properly resolved until the application of genomic studies to lager yeast. In key papers, Liti et al. (2005) and Dunn and Sherlock (2008) showed that the lager yeast could be divided into two genetically distinct groups and that these corresponded exactly with the traditional Saaz and Frohberg designations used by brewers (Glendinning 1899). Both groups contained *S. cerevisiae* and *Saccharomyces bayanus*-like DNA, though the Saaz or group 1 yeast contained proportionally more *S. bayanus*-type DNA. The Frohberg (group II) yeast contained relatively more DNA, appearing to be triploid rather than diploid based on the CGH-array analysis. Whole-genome analysis later revealed the Saaz and Frohberg groups to be triploid and tetraploid, respectively (Nakao et al. 2009; Walther et al. 2014). A further breakthrough came in 2011 with the first report of *S. eubayanus*, which had been isolated from Patagonia, where it was found associated with *Nothofagus* (Libkind et al. 2011). Genetic analysis revealed that the non-*S. cerevisiae* moiety of the lager yeast genome was almost certainly derived from *S. eubayanus*.

It has generally been assumed that the initial hybridization between the parental strains occurred when *S. eubayanus* contaminated a traditional brewery fermentation (Gibson and Liti 2015). This scenario is supported by the fact the *S. cerevisiae* component of the lager yeast genome seems to more closely resemble ale strains of *S. cerevisiae* than wild strains (Dunn and Sherlock 2008; Monerawela et al. 2015), though definitive proof is still lacking and it may be too early to discount the possibility of the progenitor being, e.g., a wild *S. cerevisiae* × *S. eubayanus* hybrid. A further assumption is that this event occurred in Central Europe in approximately the sixteenth century, based on the advent of lager brewing in this area and at this time. It is tempting to speculate that the 1533 prohibition of brewing in Bavaria during the summer months (Dornbusch, 1997) provided the conditions necessary for the competitive success of the cryotolerant *S. pastorianus* hybrid relative to traditional ale strains*.* It is, however, likely that such assumptions will be reassessed or possibly discarded as more information on the lager yeast genome and the ecology of the parental species becomes available.

Since the original isolation of *S. eubayanus* in South America, there have been a number of isolations elsewhere, including from North America (Peris et al. 2014, 2016), East Asia (Bing et al. 2014), and New Zealand (Gayevskiy and Goddard 2015) but, interestingly, not as yet from Europe. Recent sequence analysis suggests that there may be a Northern Hemisphere, or “Holarctic,” group of related strains with a wide geographical distribution, comprised (so far) of certain strains found in Tibet and in North America (Peris et al. 2016). Furthermore, it appears that it is a combination of the standing variation found among the Tibetan and North American strains that most closely matches the *S. eubayanus* parent of lager yeast (Peris et al. 2016). It therefore cannot be concluded with any certainty that the parental *S. eubayanus* strain came directly from Asia as suggested by the “Silk Road” hypothesis (Bing et al. 2014). Rather, an undiscovered population is most likely resident in Europe and individuals from this population were probably involved in the original hybridization event (or events) that gave rise to the lager yeast. A comparable situation has been observed with *S. kudriavzevii* which exists in Europe but was only found after suitable methodology was developed for its isolation (Sampaio and Gonçalves 2008; Lopes et al. 2010). Until this time, the species had only been found in hybrid form with *S. cerevisiae* in European vineyards and was only known to occur in its pure form in Asia (Naumov et al. 2000). Our understanding of the natural ecology of wild *Saccharomyces* species is severely limited (Goddard and Greig 2015) and it is probable that *S. eubayanus* (and other *Saccharomyces* species) will eventually be uncovered in Europe from an unexplored ecological niche.

Interspecific hybrids are not only used in brewing, but are commonly exploited in agriculture in order to significantly improve animal and crop yields (Chen 2013; Fu et al. 2015; Schnable and Springer 2013). This is because hybrid species often exhibit superior phenotypic qualities relative to parent strains, i.e., heterosis or hybrid vigor, and are chosen for their improved growth rates and crop yields. Phenotype amplification and heterosis have also been observed in studies on de novo yeast hybrids, which have exhibited a range of improved traits including faster fermentation rates, more complete sugar use, greater stress tolerance, and increases in aroma compound production (Bellon et al. 2011, 2013, 2015; Dunn et al. 2013; Gamero et al. 2013; Hebly et al. 2015; Krogerus et al. 2015, 2016; Mertens et al. 2015; Piotrowski et al. 2012; Plech et al. 2014; Snoek et al. 2015; Steensels et al. 2014). Interspecific hybridization can be seen as a powerful strain development tool for brewing yeast, one which enables the combination and enhancement of phenotypic features from different parent strains. Moreover, these new hybrid strains can be generated without the use of targeted genetic modification, the use of which in the brewing industry still remains limited as a result of regulations and public opinion (Twardowski and Malyska 2015). In addition to their immediate industrial applications, these new yeast hybrids may also help to elucidate the evolutionary history of industrial hybrid yeast strains, which still remains a subject of debate (Baker et al. 2015; Okuno et al. 2016; Peris et al. 2016).

This review will discuss the use of hybridization as a strain development tool for brewery applications, with particular focus on the recent research that has been carried out on de novo lager yeast. First, studies on the creation and use of hybrid yeast in brewery environments will be summarized. Then, specific industry-relevant phenotypes will be described individually. In addition, hybrid genome regulation and stability will also be discussed briefly. Finally, methods for generating interspecific yeast hybrids will be summarized and their relative merits discussed. Discussion on natural lager yeast hybrids will be kept to a minimum, as this topic has recently been reviewed elsewhere (Gibson and Liti 2015; Wendland 2014).

## Artificial hybrids

The generation of yeast hybrids, mainly for ale brewing purposes, has been carried out for decades already (Hammond and Eckersley 1984; Johnston 1965; Russell et al. 1983; Spencer and Spencer 1977). Early work involved the breeding of *S. cerevisiae* ale and laboratory strains in attempts to create intraspecific hybrids with improved fermentation rates and attenuation (Johnston 1965; Spencer and Spencer 1977). However, applying classic yeast breeding to brewing yeast is challenging, as industrial brewing strains often suffer from poor sporulation efficiencies and viabilities, presumably as a result of aneuploidy (Bilinski et al. 1986; Codón et al. 1995). Low fertility can be overcome through the use of rare mating or protoplast fusion, neither of which require the use of spores or haploid cells. These techniques have been used, e.g., to introduce dextrin fermentation from *S. cerevisiae* (syn. *S. cerevisiae* var. *diastaticus*) to both ale and lager yeast (Choi et al. 2002; Russell et al. 1983; Tubb et al. 1981), to improve the flocculation of industrial brewing strains through electrofusion (Urano et al. 1993), and to improve the ester formation and fermentation rate of ale yeast through fusion with a sake yeast (Mukai et al. 2001). More recently, selection and breeding of intraspecific hybrids with superior aroma compound production from pools of hundreds of parent strains has been accomplished using modern robot-assisted high-throughput techniques (Steensels et al. 2014).

Breeding of lager yeast has also been attempted previously, but is relatively difficult due to their aneuploidy and hybrid nature (Dunn and Sherlock 2008; Greig et al. 2002; Pfliegler et al. 2012). These result in low sporulation efficiencies, spore viabilities, and mating frequency, as was revealed by early work on Saaz-type *S. pastorianus* at Carlsberg (Gjermansen and Sigsgaard 1981). More recently, spore clones from presumably the same Saaz-type *S. pastorianus* strain were crossed with an *S. cerevisiae* ale strain to yield hybrids with improved growth at higher temperatures and resistance to high ethanol concentrations (Garcia Sanchez et al. 2012). Breeding with Frohberg-type *S. pastorianus* strains is also limited by low sporulation frequencies (Ogata et al. 2011). Again, as was previously discussed, these limitations can be overcome through the use of rare mating or protoplast fusion, which have also been successfully applied to lager yeast (Janderová et al. 1990; Russell et al. 1983; Sato et al. 2002).

Another factor that limited the breeding of lager yeast was the absence of the non-*S. cerevisiae* parent. However, the recent discovery of *S. eubayanus* (Libkind et al. 2011) has permitted the creation of novel artificial lager yeast hybrids (Alexander et al. 2016; Hebly et al. 2015; Krogerus et al. 2015, 2016; Mertens et al. 2015). These hybrids possess great potential value for the brewing industry, as it has been shown that they may ferment faster, possess a broader temperature tolerance range, and produce more diverse aroma compounds than their parent strains. Recent studies on such hybrids have been restricted mainly to hybridization with the *S. eubayanus* type strain (CBS 12357), which alone has been shown to perform poorly in wort fermentations relative to lager yeast strains (Gibson et al. 2013). Nevertheless, it does possess many traits advantageous for lager brewing, such as low-temperature growth (down to 4 °C), efficient maltose use, and production of desirable aroma compounds, which can be inherited by the hybrids (Gibson et al. 2013; Hebly et al. 2015, Krogerus et al. 2015, 2016; Mertens et al. 2015). It is expected that the diversity of new lager yeast strains will increase in the near future as new isolates of *S. eubayanus* become available for mating.

Aside from lager yeast hybrids, the use of de novo interspecific hybrids created from other species in the *Saccharomyces* genus (i.e., *Saccharomyces arboricola*, *Saccharomyces kudriavzevii*, *Saccharomyces mikatae*, *Saccharomyces paradoxus*, or *S. uvarum*) for brewing purposes has not been explored. However, recent studies on the use of de novo *S. cerevisiae* interspecific hybrids with *S. kudriavzevii* (Bellon et al. 2011; Lopandic et al. 2016), *S. mikatae* (Bellon et al. 2013), *S. paradoxus* (Bellon et al. 2011), and *S. uvarum* (Bellon et al. 2015; Lopandic et al. 2016) for wine making have revealed the potential for increasing aromatic diversity and fermentation performance. As many of these “alternative” *Saccharomyces* species are also cold-tolerant, e.g., *S. kudriavzevii* and *S. uvarum* (Gonçalves et al. 2011; López-Malo et al. 2013; Paget et al. 2014), they represent feasible alternatives to *S. eubayanus* in interspecific hybrids for lager brewing purposes and may compensate for the current paucity of *S. eubayanus* isolates. Another group of hybrids, that currently remains poorly explored in relation to brewing applications, is the intergeneric hybrid group. Such hybrids have been successfully constructed through protoplast fusion (Lucca et al. 2002; Spencer et al. 1983). Species belonging formerly to the *Saccharomyces* sensu lato group may be of particular interest in this regard. Interest in the potential of non-*Saccharomyces* yeasts in brewing has increased in recent years (Basso et al. 2016; Canonico et al. 2016; Michel et al. 2016). However, it remains to be seen if such strain development approaches will find acceptance for industrial-scale brewing.

In brief, de novo yeast hybrids have been used successfully to improve beer fermentation in a number of respects, including fermentation rate, aroma formation, and stress tolerance. The following section will discuss in more detail how various phenotypes important for beer fermentation can be affected in these yeast hybrids. A list of recent and relevant studies investigating the use of de novo yeast hybrids for beer fermentation has been compiled in Table 1.

**Table 1:** A summary of studies published since the year 2000 investigating the use of de novo yeast hybrids in beer fermentation

Parental strains		Key results	Reference
*S. cerevisiae* ale strain	*S. cerevisiae* sake strain	The hybrid had an increased fermentation rate and produced increased concentrations of certain aroma compounds	Mukai et al. 2001
*S. cerevisiae* ale strain	*S. cerevisiae* strain (syn *S. cerevisiae* var. *diastaticus*)	Hybrids had higher attenuation levels (i.e., utilized a higher ratio of the original wort carbohydrates) and ethanol yield than the brewing parent strain	Choi et al. 2002
*S. cerevisiae* ale strain	Cold-tolerant *S. bayanus* strain	Hybrids had greater fermentation rates than the ale parent in low temperature wort fermentations	Sato et al. 2002
*S. cerevisiae* ale strain	Saaz-type *S. pastorianus* strain	Hybrids showed improved osmo- and temperature tolerance and fermentation performance compared to the lager parent strain	Garcia Sanchez et al. 2012
Various *S. cerevisiae* ale, bakery, sake, and wine strains		Hybrids with higher acetate ester formation than the parent strains were attained. Best-parent heterosis with regards to aroma formation was more common in outbred hybrids than in inbred hybrids	Steensels et al. 2014
*S. cerevisiae* laboratory strain	*S. eubayanus* type strain	The hybrid had improved sugar utilization and fermentation rate compared to the parent strains in synthetic wort	Hebly et al. 2015
*S. cerevisiae* ale strain	*S. eubayanus* type strain	Hybrids exhibited increased fermentation rates and aroma compound formation compared to parent strains	Krogerus et al. 2015
Various *S. cerevisiae* ale and wine strains	*S. eubayanus*	Hybrids produced a greater diversity of aroma compounds compared to traditional lager yeast and parent strains	Mertens et al. 2015
*S. cerevisiae* ale strain	*S. eubayanus* type strain	Hybrids exhibited increased fermentation rates and aroma compound formation compared to parent strains. Fermentation performance and aroma formation of the hybrids increased with ploidy. The aroma profile of de novo lager yeast hybrids can be controlled based on the relative contribution of parental DNA	Krogerus et al. 2016

## Hybrid phenotypes

### Aroma production

During wort fermentation, yeast produce a range of metabolites which contribute to beer aroma. The main groups of yeast-derived aroma-active compounds in beer are higher alcohols, esters, sulfur compounds, volatile phenols, vicinal diketones, and aldehydes (for recent reviews, see Krogerus and Gibson 2013; Landaud et al. 2008; Pires et al. 2014; Vanderhaegen et al. 2006). However, not all yeast-derived aroma compounds are desirable. Thus, the target of many strain development strategies is to increase the production of certain aroma compounds, such as esters, while decreasing the production of off-aromas, such as vicinal diketones and sulfur compounds. Hybridization offers a valuable alternative, as diverse aroma phenotypes can be combined, and increased aroma formation can be achieved through best-parent heterosis.

Studies on yeast hybrids in beverage fermentation have revealed the possibility of either increasing aroma production or achieving mid-parent values in hybrids (Bellon et al. 2011, 2013; da Silva et al. 2015; Gamero et al. 2013; Krogerus et al. 2015, 2016; Mertens et al. 2015; Mukai et al. 2001; Steensels et al. 2014). Early work by Mukai et al. (2001) showed that the concentrations of 2-methylpropyl acetate (fruit aroma) and ethyl hexanoate (apple/aniseed aroma) in beer could be increased by using an ale × sake intraspecific hybrid compared to the ale parent strain. More recently, a large-scale breeding study by Steensels et al. (2014) revealed that a 45 % increase in 3-methylbutyl acetate (banana aroma) formation could be achieved in intraspecific hybrids. Outbred hybrids, i.e., those formed by hydridization between spores (segregants) from two different parent strains, in particular tended to show a greater increase in ester production compared to inbred hybrids, i.e., those formed by hydridization between spores (segregants) derived from a single parent strain. It has been shown that heterosis in regard to the growth rates of yeast hybrids formed from domesticated parent strains is positively correlated with sequence divergence (Plech et al. 2014; Shapira et al. 2014). However, Steensels et al. (2014) did not see an increase in 3-methylbutyl acetate formation as the genetic distance of the parent strains was increased. Nevertheless, it is possible that this effect is more pronounced in interspecific hybrids compared to intraspecific hybrids as suggested, e.g., in the study by da Silva et al. (2015) with regard to ethyl ester formation.

**Fig. 1:**
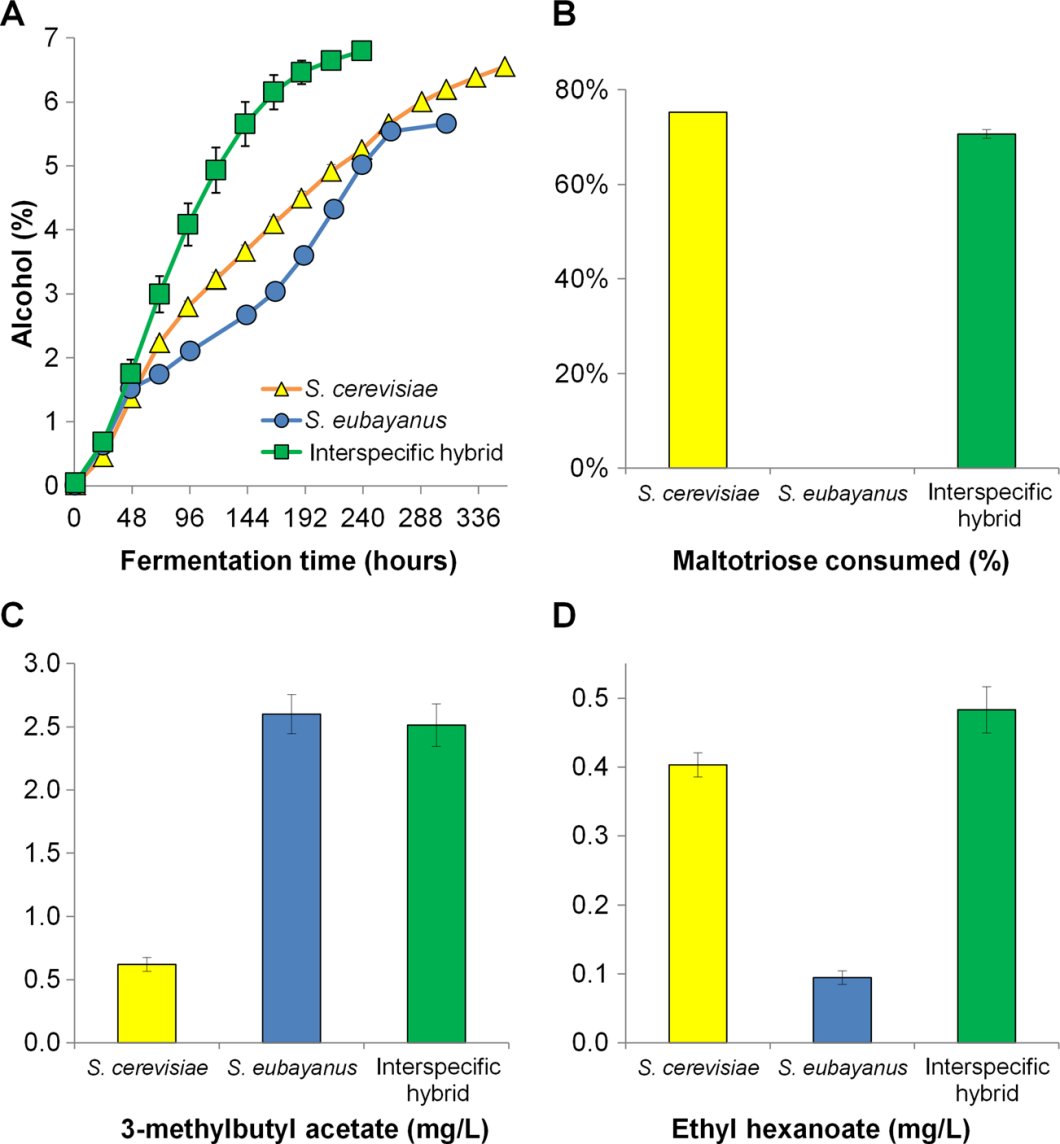
The **a** alcohol content, **b** percentage of maltotriose consumed, **c** final 3-methylbutyl acetate concentration (mg/L), and **d** final ethyl hexanoate concentration (mg/L) of a 15 °P all-malt wort fermented at 15 °C with an *S. cerevisiae* A81062 ale strain, the *S. eubayanus* C12902 type strain, and an allotetraploid interspecific lager hybrid (hybrid C4) between the two. *Values* are means from two independent fermentations and *error bars* where visible represent the standard deviation. A solution of *X* °P has the same density as an aqueous sucrose solution containing *X* g of sucrose in 100 g of solution. The figure was recreated using data from Krogerus et al. (2016)

Probably due to the close relatedness of natural lager yeast hybrids (Dunn and Sherlock 2008; Okuno et al. 2016), a limited aroma spectrum exists within this group (Gibson et al. 2013; Mertens et al. 2015). It was recently shown that this diversity can be improved by generating new interspecies lager hybrids (Mertens et al. 2015). Results revealed that the aroma profiles of these strains ranged from worst- to best-parent levels, with several of the hybrids producing higher concentrations of aroma compounds than either of their parents (especially 3-methylbutyl and 2-methylpropyl acetate). A similar result was obtained in other work (Krogerus et al. 2015), where de novo lager hybrids produced beers with higher overall concentrations of esters compared to the parent strains. Certain esters, such as ethyl hexanoate, were formed at higher concentrations than either parent strain, and above the flavor threshold. In a follow-up study, during which hybrids with different ploidy from the same parent strains were compared, it was further shown that the aroma profile of hybrids can be controlled based on the relative contribution of parental DNA (Krogerus et al. 2016). The highest concentrations of ethyl and acetate esters were produced by the tetraploid hybrid (Figure 1), while the triploid hybrid (containing proportionally more of the *S. cerevisiae* parent genome) formed lower amounts of acetate esters which were associated with the *S. eubayanus* parent strain. Transcriptional analysis and copy number estimation of several key genes related to the synthesis of these ethyl and acetate esters suggested that these observed differences can be partly attributed to higher gene copy numbers and transcription levels at higher ploidy. It was recently revealed that orthologous alcohol acetyltransferases (i.e., Atf1 and Atf2) derived from various *Saccharomyces* species show differences in their functional properties (Stribny et al. 2016), which may also contribute to the more diverse aroma formation that has been observed in de novo lager hybrids.

While hybridization can be used to modify the production of desirable aroma compounds, one must be aware of the inherent risk of simultaneously increasing the formation of undesirable off-flavors. The formation of ethyl acetate, which is unpleasant at high concentrations, is positively correlated with 3-methylbutyl acetate formation, and thus hybrids with increased levels of the latter tend to show higher levels of the former (Steensels et al. 2014). The formation of the unwanted vicinal diketone diacetyl may also increase in hybrid strains compared to their parents (Krogerus et al. 2016). However, hybridization can also be used to decrease or completely remove unwanted aroma compounds associated with one of the parent strains. For instance, Tubb et al. (1981) removed the ability to produce 4-vinylguaiacol (phenolic off-flavor) from a dextrin-fermenting *S. cerevisiae* strain by first mating it with an ale strain and then screening meiotic segregants of this hybrid for low 4-vinyl guaiacol production. The phenolic off-flavor phenotype has been attributed to functional *PAD1* and *FDC1* genes (Mukai et al. 2014), and recent whole-genome sequencing of industrial brewing strains has revealed that strains lacking this phenotype contain loss-of-function mutations in either of these genes (Gallone et al. 2016; Gonçalves et al. 2016). Gallone et al. (2016) also demonstrated that hybrid strains lacking the ability to produce 4-vinyl guaiacol can be constructed if both of the parent strains contain such loss-of-function mutations in either *PAD1* or *FDC1*. Similarly, Bizaj et al. (2012) were able to decrease the formation of H_2_S (rotten egg aroma) in hybrid wine strains by mating them with a low H_2_S-producing *S. cerevisiae* wine strain. We have also observed that the *S. eubayanus* type strain, which has been used to create the majority of de novo lager hybrids, can produce sulfuric off-aromas (e.g., ethanethiol and ethyl thioacetate) during wort and must fermentations (our unpublished data). These traits may also transfer to any hybrids formed from this strain. However, such hybrids tend to produce decreased mid-parent concentrations of these unwanted sulfuric compounds (our unpublished data). In conclusion, hybridization can be used as a means to increase the production of desirable aroma compounds and decrease the production of unpleasant volatiles relative to the parent strains.

### Temperature tolerance

The ability to tolerate low temperatures is one of the defining characteristics of lager yeast and permits the low-temperature fermentation necessary for production of lager beer. This style is characterized by a clean aroma relative to the more intense fruit and floral notes characteristic of ales, a difference that is mostly due to the different fermentation temperatures employed. Low-temperature lager fermentations require the yeast to be able to survive and stay metabolically active in the cold (Gibson and Liti 2015). It is known that the cold tolerance of *S. pastorianus* is a result of its hybrid nature. However, the mechanisms by which this yeast and its cold-tolerant parent *S. eubayanus* cope with low temperatures are not known. As *S. eubayanus* has only recently been discovered, we have a limited understanding of the metabolic processes responsible for its superior cryotolerance. Gibson et al. (2013) showed with lager yeast that the more dominant the *S. eubayanus* genome portion is, the more cold-tolerant the strain is. For instance, Saaz-type strains are better adapted to cold than Frohberg-type. The presence of α-glucoside transporters that function better at lower temperatures, such as Mtt1, could play a role in the yeast performance at these temperatures (Vidgren et al. 2010 2014; Magalhães et al. 2016). Artificial interspecific hybrids of *S. cerevisiae* and *S. eubayanus* strains clearly have the ability to efficiently ferment wort at temperatures as low as 12 °C (Krogerus et al. 2015; Hebly et al. 2015; Mertens et al. 2015). How the hybrid genomes cooperate to produce this kind of phenotype is not yet clear.

In addition to *S. eubayanus*, other members of the *Saccharomyces* genus, such as *S. kudriavzevii* and *S. uvarum,* are also adept at growing and fermenting at low temperatures (González et al. 2006; Masneuf-Pomarède et al. 2010). These species are usually associated with wine and cider fermentation (González et al. 2006; Naumov et al. 2001). In the case of *S. kudriavzevii*, only interspecific hybrids have been found in fermentation conditions (Sampaio and Gonçalves 2008; Lopes et al. 2010). Cold-tolerant *S. uvarum* strains show higher ethanol sensitivity in wine fermentations at warmer temperatures (25 °C) than they do at low temperatures (13 °C), possibly due to a different fatty acid composition of the cell (Kishimoto et al. 1994; Masneuf-Pomarède et al. 2010). We have observed a similar behavior from the *S. eubayanus-*type strain, with it being sensitive to ethanol at warm temperatures but not affected in the cold (unpublished data). The response of these species to the combined effect of temperature and ethanol in the cell membrane deserves further investigation.

Low temperature is known to affect the efficiency of protein translation, fluidity of the membrane, lipid composition, protein folding, stability of messenger RNA (mRNA) structures and enzymatic activities (Aguilera et al. 2007; Sahara et al. 2002; Schade et al. 2004; Tai et al. 2007). Salvadó et al. (2011) showed, prior to the discovery of *S. eubayanus*, that *S. kudriavzevii* had the lowest optimal growth temperature of all the *Saccharomyces* species. Gonçalves et al. (2011) compared the rate of adaptation between *S. cerevisiae* and *S. uvarum* and found that groups of genes associated with cell wall mannoproteins, ribosomal stalk, translation elongation factors, and glycolysis have undergone “accelerated” evolution. Paget et al. (2014) identified genes associated with glycerol and acetaldehyde metabolism as being responsible for the cryotolerance of *S. kudriavzevii* and were able to replicate this effect by overexpressing the genes in *S. cerevisiae*. García-Ríos et al. (2016) further observed that *S. kudriavzevii* is better adapted to grow at low temperatures due to more efficient protein translation. This is true also for cold-adapted *S. cerevisiae* strains (Salvadó et al. 2016). None of these studies has however included *S. eubayanus*, and although similar mechanisms may be involved in the cold tolerance of this species, different species are known to react differently to variations in temperature. For example, *S. uvarum* improves respiration rates at low temperatures while *S. kudriavzevii* has superior ethanol production under similar conditions (Gonçalves et al. 2011).

Lager yeast hybrids clearly benefit from the cryotolerance conferred by *S. eubayanus*. The exceptional cold tolerance of this species is illustrated by the fact that even a cold-tolerant species like *S. uvarum* can benefit from the relationship. Almeida et al. (2014) have shown that domesticated strains of *S. uvarum*, i.e., those used in low-temperature cider and wine fermentations, contain introgressed DNA from *S. eubayanus*. Such introgressions are typically absent in wild strains of *S. uvarum* and the genetic contribution from *S. eubayanus* appears to be the main differentiating factor between wild and domesticated strains of the species. The origin of this genetic material has yet to be determined, i.e., directly from a natural population of *S. eubayanus* or indirectly via interaction with an existing *S. eubayanus* hybrid.

### Sugar utilization

Maltose and maltotriose are the main fermentable sugars of wort. The parent strains of lager hybrids have different sugar utilization characteristics. Brewing strains of *S. cerevisiae* are usually able to utilize both maltose and maltotriose efficiently, whereas *S. eubayanus* appears able to utilize only maltose (Gallone et al. 2016; Gibson et al. 2013; Hebly et al. 2015). This seems to be due to a lack of maltotriose transporters (Hebly et al. 2015; Baker et al. 2015). However, so far, only one Patagonian isolate, the *S. eubayanus* type strain CBS12357, has been characterized in terms of maltotriose utilization (Gibson et al. 2013; Hebly et al. 2015) and newly found strains, e.g., Northern Hemisphere isolates from China and North America (Bing et al. 2014; Peris et al. 2016) and the New Zealand isolate (Gayevskiy and Goddard 2016) remain to be characterized. The two subgroups of the lager yeasts, Saaz and Frohberg, also differ in their sugar utilization characteristics. Frohberg strains can utilize both maltose and maltotriose, whereas the more *S. eubayanus*-like Saaz strains are in general unable to ferment maltotriose, resulting in lower growth and fermentation rates (Gibson et al. 2013; Magalhães et al. 2016). This might be because Saaz strains lost significant portions of their *S. cerevisiae* genome after hybridization (Dunn and Sherlock 2008; Walther et al. 2014) and possibly lost genes needed for maltotriose utilization during this reorganization of the genome. On the other hand, some authors suggest that Saaz and Frohberg lineages were created by two distinct hybridization events between different ale strains (Dunn and Sherlock 2008; Baker et al. 2015; Monerawela et al. 2015) or even between different *S. eubayanus* strains (Baker et al. 2015) possibly possessing different maltotriose utilization genes, which might explain differences in maltotriose utilization seen between the groups.

In general, at the low temperatures used for lager brewing (8–15 °C), de novo lager hybrids outperform the parental strains in terms of maltose and, especially, maltotriose utilization rates (Krogerus et al. 2015, 2016; Mertens et al. 2015). De novo interspecific hybrids have even displayed similar fermentation efficiencies to *S. pastorianus* strains currently used for commercial beer production (Krogerus et al. 2015; Mertens et al. 2015). The majority of the 31 interspecific lager hybrids created by Mertens et al. (2015) outperformed the parental strains in regards to ethanol production during fermentations at 16 °C. Three of the hybrids (all from different *S. cerevisiae* parents crossed with the Y567 Patagonian isolate of *S. eubayanus*) showed an ethanol production capacity similar or higher to the best reference *S. pastorianus* strains. The difference in ethanol production between strains was shown to be largely due to the ability to efficiently ferment maltotriose present in the wort. Strains producing less than 5 % alcohol by volume only fermented 50–60 % of the available maltotriose, whereas strains producing more than 5 % ethanol fermented up to 70 % of the maltotriose.

In the studies of Krogerus et al. (2015, 2016), lager hybrids resulting from a cross between an ale strain and the *S. eubayanus* type strain were also observed to ferment more efficiently than the parental strains (Figure 1). All hybrids had inherited the maltotriose uptake ability of the *S. cerevisiae* parent as well as the cold tolerance of *S. eubayanus,* enabling successful growth and fermentation at low temperatures (12 and 15 °C). The sugar profiles of the original wort and the beers produced revealed that the greater sugar uptake relative to the *S. eubayanus* parent was a result of efficient maltotriose utilization. The *S. cerevisiae* parent had only limited ability to utilize wort sugars at lower temperatures and there was a significant amount of residual maltose in the resulting beer. Krogerus et al. (2016) also revealed that the ploidy of de novo lager hybrids influences fermentation performance, as the hybrid strains with higher DNA content (i.e., the allotetraploid hybrid followed by the allotriploid hybrid) were clearly superior to lower ploidy hybrids in the fermentation of wort at 15 °C. There was a clear link between fermentation performance of hybrids and different sugar consumption abilities during fermentation, as strains fermenting fastest also consumed maltose and maltotriose fastest. As it is the uptake of maltose and maltotriose that tends to limit fermentation capacity during brewing (Alves et al. 2007; Rautio and Londesborough 2003), higher ploidy of hybrids would provide for a greater number of maltose/maltotriose transporter genes in hybrid genomes, which could account for improved uptake of these sugars. The interspecific hybrid between *S. eubayanus* type strain and the *S. cerevisiae* IMK439 laboratory strain studied by Hebly et al. (2015) also inherited the cryotolerance of *S. eubayanus* and maltotriose utilization ability of the *S. cerevisiae* parent. Additionally, it was able to grow more rapidly on maltose at 20 °C, resulting in a fermentation time that was 10 h shorter compared to *S. cerevisiae* parent.

So far, only the Patagonian *S. eubayanus* isolates and, in particular, the type strain CBS12357, have been used as the non-*S. cerevisiae* parent in *S. cerevisiae* × *S. eubayanus* crosses (Hebly et al. 2015; Krogerus et al. 2015, 2016; Mertens et al. 2015). As *S. eubayanus* apparently cannot use maltotriose, industrial lager strains seem to have inherited this trait from the original *S. cerevisiae* parent. However, as discussed in the “Introduction” section, results from whole-genome sequencing of recently discovered *S. eubayanus* strains have shown that isolates from the Northern hemisphere, North America and Tibet in particular, seem to be the closest relatives to the domesticated *S. eubayanus* half of the lager hybrid (Bing et al. 2014; Peris et al. 2016). This raises the question of whether there may exist *S. eubayanus* lineages capable of maltotriose uptake. Interestingly, analysis of the genome sequence of the Tibetan *S. eubayanus* isolate (Bing et al. 2014) identified ORFs that exhibited better similarity with *AGT1* than with *MAL31* (Hebly et al. 2015) suggesting that the Tibetan *S. eubayanus* might actually possess a maltotriose transporter gene (*AGT1*) found to be missing from the complete genome assembly of the Patagonian *S. eubayanus* strain (Baker et al. 2015). However, the ability of newly isolated North American strains and of the Tibetan strain to grow on maltotriose remains to be assessed (Bing et al. 2014; Peris et al. 2016).

Aside from fermentable sugars such as maltose and maltotriose, wort contains a large share of non-fermentable carbohydrates, the most abundant of which is dextrin. Its utilization during fermentation would result in higher ethanol yields and lower-carbohydrate beer. Some strains of *S. cerevisiae* (syn. *S. cerevisiae* var. *diastaticus*) have been shown to ferment dextrin, and this ability has been transferred to both ale and lager yeast through hybridization (Choi et al. 2002; Russell et al. 1983; Tubb et al. 1981). These hybrids showed higher fermentation degrees and ethanol yields than the brewing yeast parents.

## Hybrid genome function and stability

It might be expected that in newly formed interspecific hybrids, there is a certain level of functional disorder due to the clash of different regulatory networks, and consequently, that this disorder has an influence on the evolution of the genome. Relatively little is known of the transregulation of gene activity in de novo hybrids. Bolat et al. (2013) have shown that removal of the *S. eubayanus* allele of the regulator *ARO80* from a production strain of *S. pastorianus* did not significantly affect expression of the *S. eubayanus* form of the target gene *ARO10*. Results suggested that the *S. cerevisiae* regulator could compensate entirely for the loss and, that at least in natural *S. pastorianus* strains, cooperative mechanisms exist between subgenomes. Proteome and transcriptome studies have, however, shown that significant differences in subgenome activity can occur in lager yeast (Caesar et al. 2007; Horinouchi et al. 2010; Minato et al. 2009; Yoshida et al. 2007), suggesting that gene regulation in interspecies hybrids is not seamlessly integrated across subgenomes. Gibson et al. (2015) showed that differences in expression of the two different alleles of the regulatory gene *ILV6* in *S. pastorianus* could influence strain phenotypes (in this case, the production of α-acetolactate). Otherwise, there was no functional difference between the gene products as determined by their over-expression with the same promoter. In other cases, functional divergence has been observed for many gene products that influence brewing properties (Iijima and Ogata 2010; Ogata et al. 2013; He et al. 2014). Similar investigations must be carried out with newly created hybrids to determine the level of initial regulatory disorder and also how regulatory issues are resolved over time. Tirosh et al. (2009) measured gene activity in de novo *S. cerevisiae* × *S. paradoxus* hybrids and found that both cis- and transregulation could be observed and that this was influenced by the environmental conditions to which the hybrids were exposed. It would be of interest to determine how subgenome activity in new *S. cerevisiae* × *S. eubayanus* hybrids is influenced by environmental conditions, particularly those conditions, such as temperature extremes, that have the greatest differential impact on the physiology of the parent strains. Wendland (2014) has suggested that differences in fermentation temperature may have molded the genomes of the Saaz and Frohberg strains, which display differences in their tolerance to low-temperatures (Gibson et al. 2013; Walther et al. 2014). This hypothesis could be tested by adapting cultures of the same hybrid strain in parallel to either high or low temperatures and assessing the genetic changes occurring in each case.

Extensive chromosome loss and intrachromosomal translocations, sequence divergence, and chromosome copy number variation in the genomes of lager yeast (van den Broek et al. 2015) indicate that the *S. pastorianus* genome is inherently unstable. Such instability is not unexpected given the high level of regulatory incompatibilities (Landry et al. 2007) and functional redundancy that are associated with polyploid hybrids (Kumaran et al. 2013; Selmecki et al. 2015). The lager yeast genome is certainly amenable to change via evolutionary engineering, which has been applied to improve stress tolerance (Blieck et al. 2007; Ekberg et al. 2013; Huuskonen et al. 2010; James et al. 2008) and modify beer flavor profile (Mikkelsen et al. 1979; Strejc et al. 2013). As the possibility of creating artificial lager hybrids has existed for only a short time, we have limited information about the stability or adaptability of newly formed genomes. Previous research has indicated that one subgenome in a laboratory-made hybrid is often more susceptible to change or elimination. This was the case for example with *S. cerevisiae* × *S. uvarum* hybrids, where the *S. uvarum* moiety was gradually reduced after successive meiotic (Antunovics et al. 2005) or mitotic (Masneuf-Pomarède 2007; Sebastiani et al. 2002) divisions. Likewise, Lopandic et al. (2016) noted the loss of *S. kudriavzevii* chromosomes from an artificial *S. cerevisiae* × *S. kudriavzevii* hybrid, particularly after these fertile hybrids underwent meiosis. A similar reduction in *S. kudriavzevii* DNA has been observed in natural wine and beer hybrids (González et al. 2008; Peris et al. 2012). There is evidence that this differential DNA loss can be due to environmental conditions. Piotrowski et al. (2012) showed that during adaptation to high temperature, there was a progressive loss of *S. uvarum* chromosomes from a laboratory *S. cerevisiae* × *S. uvarum* hybrid. This suggests that parental physiology may direct the evolution of the hybrid genome with, in this example, progressive loss of the cold-tolerant subgenome at high temperature, leaving a greater proportion of the high-temperature-tolerant *S. cerevisiae* DNA. Whether this can explain the greater contribution of *S. eubayanus* DNA in the cold-tolerant Saaz lager yeast remains to be seen.

## Hybrid generation

**Fig. 2:**
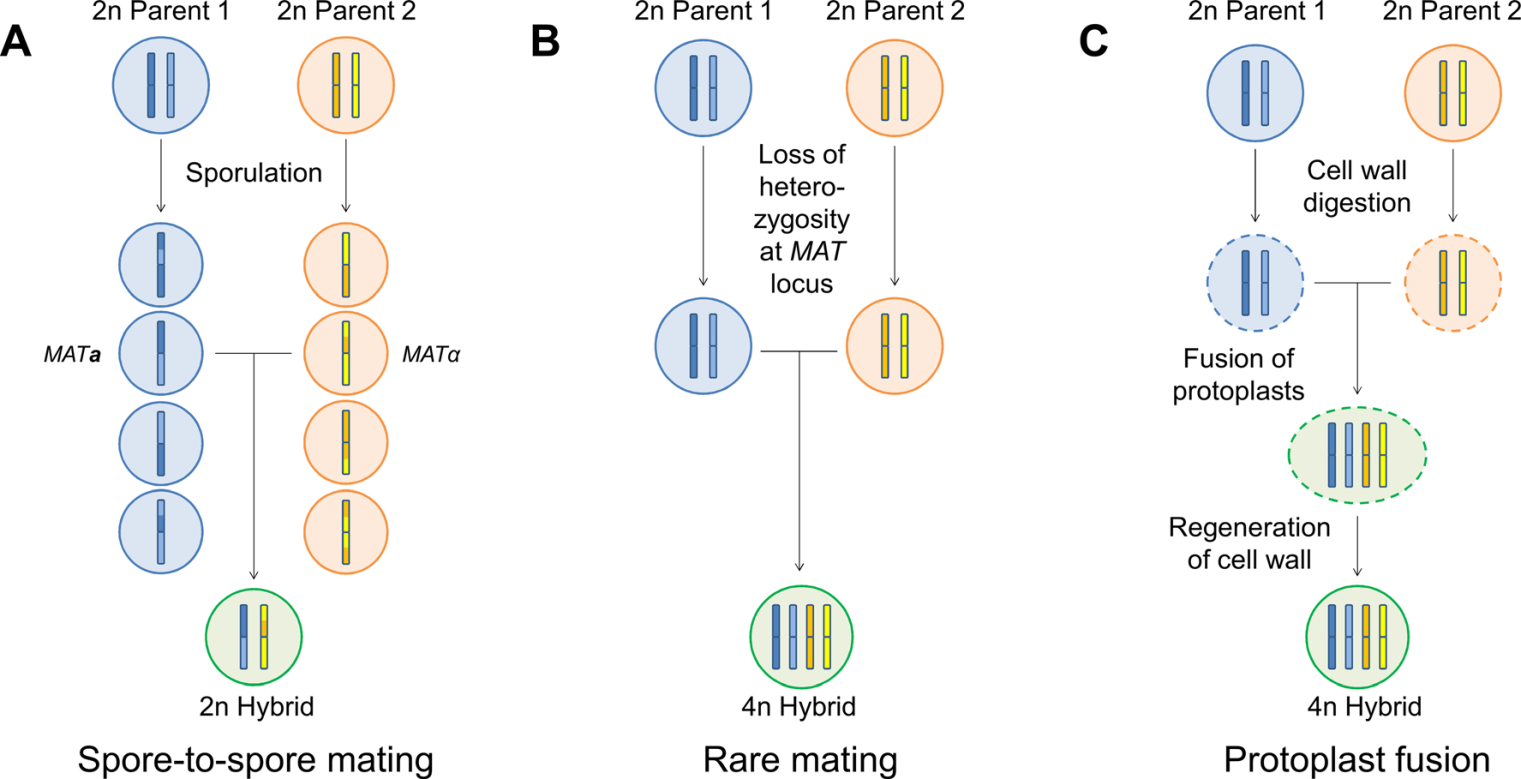
An overview of different hybridization methods. During **a** spore-to-spore mating, the diploid (2n) parent strains are first sporulated, after which haploid spores of opposite mating type derived from the two parent strains are brought together and allowed to mate. A diploid (2n) hybrid is formed. During **b** rare mating, the diploid (2n) parent strains are brought together without any prior sporulation. The cells are not able to directly mate, but rare spontaneous loss of heterozygosity at the mating-type locus can occur in a fraction of the population. As a result, diploid cells with a single mating type, which are able to mate, are formed. A tetraploid (4n) hybrid is formed. During **c** protoplast fusion, the cell walls of the diploid (2n) parent strains are first digested, after which the protoplasts are brought together and undergo fusion, followed by the regeneration of the cell wall. A tetraploid (4n) hybrid is formed

*Saccharomyces* hybrids can be generated through a variety of methods, including spore-to-spore mating, mass mating, rare mating, and protoplast fusion among others (Figure 2). Here, these methods will be discussed briefly together with an assessment of their advantages and disadvantages. Sexual hybridization occurs when haploid cells of opposite mating type (*a* or *α*) meet and fuse (for a recent review on the subject see Merlini et al. (2013)). The traditional approach to yeast breeding is through the mating of cells derived from spores. Spores from the two parent strains can be placed adjacent to one another on an agar plate with the aid of a micromanipulator, i.e., spore-to-spore mating, or randomly mixed together on solid or in liquid growth media, i.e., mass mating. These techniques have been used in the majority of the studies listed in Table 1 (Hebly et al. 2015; Krogerus et al. 2016; Mertens et al. 2015; Sanchez et al. 2012). These approaches have several advantages, including high hybridization frequencies, possible use without selection markers (with spore-to-spore mating), and typically greater genetic stability in the resulting hybrids. However, the parent strains must be able to produce viable spores and physiological traits may be lost or altered through meiotic recombination during spore formation. In the case of mass mating, selection markers (e.g., auxotrophies) or other screening methods are also required to isolate hybrids from the population of parent cells. Steensels et al. (2014) utilized a variant of this approach, where parent strains were first screened for heterothallism (i.e., the spore clones exhibit a stable mating type, and thus do not self-mate) prior to mating. Hybrid status of any isolates can be confirmed through various PCR (e.g., using ITS, interdelta or species-specific primers) or karyotyping techniques (e.g., pulsed-field gel electrophoresis) (Fernández-Espinar et al. 2000; Legras and Karst 2003; Muir et al. 2011).

If either or both of the parent strains one wishes to hybridize are unable to form viable spores, one can apply rare mating. Diploid (or higher ploidy) strains generally have *a*/*α* mating type (i.e., a heterozygous mating type locus) and do not directly mate. However, spontaneous loss of heterozygosity at the mating type locus can occur at low frequencies (10^−4^), resulting in the formation of diploid (or higher) cells with *a* or *α* mating types (Hiraoka et al. 2000). These cells may then mate to form polyploid hybrids, which may contain more or less the full genomes of both parent strains. This approach has also been used in many of the studies listed in Table 1 (Choi et al. 2002; Krogerus et al. 2015, 2016; Sato et al. 2002). The most recent of these studies (Krogerus et al. 2016) suggested that higher ploidy lager hybrids produced through rare mating outperformed (in regards to fermentation rate and aroma formation) a diploid lager hybrid formed through spore-to-spore mating. However, as the name implies, these matings occur rarely and the hybridization frequencies are typically low. Furthermore, because of the low mating frequency, selection markers (e.g., auxotrophies) are required to isolate hybrids from the population of parent cells. The genomes of hybrids formed from rare mating also tend to be less stable than those formed from mating of spores (Pérez-Través et al. 2012).

To overcome the disadvantages of low hybridization frequencies and requirement for selection markers, various strategies have been developed. Alexander et al. (2016) describe a technique that can be used to force mating type change in diploid cells by transformation with a plasmid carrying the *HO* gene under the control of an inducible promoter (this gene is repressed in *a*/*α* diploid cells). Expression of *HO* in *a*/*α* diploid cells results in mating type change to *a*- or *α*-type, allowing for rare mating with higher hybridization frequencies. The plasmids also carry drug-resistance markers, which allow for the selection of hybrids. Fukuda et al. (2016) describe another approach for selecting diploid cells with either an *a* or *α* mating type. In their technique, *a*/*α* diploid cells are transformed with a plasmid carrying either the *a*1 or *α*2 gene from the mating-type locus together with drug-resistance markers with promoters specific to either the *a*1 or *α*2 gene products. When cells containing these plasmids are grown on media containing the particular drug, only cells with an *a* or *α* mating type are able to grow (as the *a*1-*α*2 dimer will repress the transcription of the drug-resistance gene). Hence, this technique also increased the hybridization frequency of rare mating. However, both these techniques require the transformation of cells with plasmids carrying drug-resistance markers. These plasmids are easily lost from the yeast though, resulting in cells with no exogenous DNA remaining.

The final approach to generating hybrids that will be discussed in this review is protoplast fusion. With this approach, the cell walls of the parent strains are first digested (i.e., protoplasts are formed), after which the cells or protoplasts are brought together and undergo fusion, followed by the regeneration of the cell wall (van Solingen and van der Plaat 1977). As with rare mating, this technique is particularly advantageous for mating strains that rarely form viable spores. Furthermore, protoplast fusion allows for the mating of sexually incompatible cells, e.g., in the formation of intergeneric hybrids (Lucca et al. 2002). Of the studies listed in Table 1, only Mukai et al. (2001) used protoplast fusion. The disadvantages of protoplast fusion are low hybridization frequencies, the need for selection markers, and typically low genome stability in the resulting hybrids. Also, hybrids resulting from protoplast fusion may be considered genetically modified in some regions of the world.

## Future prospects and concluding remarks

The interspecific yeast hybrid *S. pastorianus* already plays a vital role in the modern brewing industry, but its phenotypic potential is limited due to it containing genetic material from only two or three individual yeast strains. To overcome this, the creation of novel brewing yeast hybrids has been shown to be a promising strain development tool for brewing yeast. Hybridization enables the combination and enhancement of a range of phenotypic features from different and diverse parent strains, and the technique has already been used to create yeast hybrids with faster fermentation, more complete sugar use, greater stress tolerance, and more diversified aroma compound production. However, the use of hybridization to improve on several other phenotypic traits still remains unexplored. These include encouraging the formation of antioxidants to increase flavor stability, decreasing the formation of unwanted off-flavors to enhance beer quality, and increasing glycerol formation for better mouthfeel in low-alcohol beer. Furthermore, studies on the use of de novo hybrids for brewing purposes have been mainly limited to hybrids created with *S. cerevisiae* or *S. eubayanus* strains as parents. Many other species in the *Saccharomyces* genus possess traits desirable for brewing, including cold tolerance and high ester formation, and thus represent feasible alternatives to *S. eubayanus* in interspecific hybrids for lager brewing purposes.

While hybrids possess various enhanced phenotypes in comparison to the parent strains, the molecular mechanisms that control and contribute to the hybrid phenotypes are not fully understood. These phenotypes include the cold and stress tolerance of lager hybrids and heterosis effect observed for aroma formation. With the greater application of sequencing in hybrid studies, it is expected that questions regarding the stability of hybrid genomes, subgenome cooperation and regulation, and the evolutionary history of *S. pastorianus* will become clearer in the future. There is also the potential for exploiting the inherent instability of hybrid genomes for advanced strain development through adaptive evolution. Hybridization can therefore be explored and utilized further in several ways, yielding powerful and diverse yeast strains for the brewing industry.
